# Growth and gut comfort of healthy term infants exclusively fed with a partially hydrolysed protein-based infant formula: a randomized controlled double-blind trial

**DOI:** 10.3389/fped.2024.1328709

**Published:** 2024-05-17

**Authors:** Paris Kantaras, Anna Kokkinopoulou, Jeske H. J. Hageman, Maria Hassapidou, Odysseas Androutsos, Maria Kanaki, Ingeborg Bovee-Oudenhoven, Eva Karaglani, Aikaterini-Maria Kontochristopoulou, Rolf Bos, Yannis Manios

**Affiliations:** ^1^ Department of Nutrition and Dietetics, School of Health Science and Education, Harokopio University, Athens, Greece; ^2^ Department of Nutritional Sciences and Dietetics, School of Health Sciences, International Hellenic University, Thessaloniki, Greece; ^3^ FrieslandCampina, Amersfoort, Netherlands; ^4^ Lab of Clinical Nutrition-Dietetics, Department of Nutrition and Dietetics, School of Physical Education, Sport Science and Dietetics, University of Thessaly, Trikala, Greece; ^5^ Institute of Agri-Food and Life Sciences, Hellenic Mediterranean University Research Centre, Heraklion, Greece

**Keywords:** infant formula, cow's milk protein hydrolysate, growth, partially hydrolysed formula, anthropometry, gut comfort

## Abstract

**Objective:**

This study aimed to investigate growth and gut comfort of healthy infants fed with a partially hydrolysed cow's milk protein-based infant formula (pHF) compared to a standard intact cow's milk protein-based formula (IPF).

**Methods:**

A double-blind, multi-center, randomized, controlled trial was performed. Healthy full-term, exclusively formula-fed infants (*n* = 345), aged ≤28 days were allocated to consume either a pHF (*n* = 173) or an IPF (*n* = 172) until the age of 17 weeks. The primary outcome was equivalence of weight gain (g/d) until the age of 17 weeks. The secondary outcomes were equivalence of other growth parameters, i.e., infants’ weight, length, head circumference, body mass index (BMI) and anthropometric *Z*-scores, while tertiary outcomes were gut comfort, formula intake, and adverse events (AEs).

**Results:**

Overall, 288 infants completed the study (pHF group: 138, IPF group: 150). No differences were observed between the two groups in weight gain (g/d) during the three-months intervention [*p* = 0.915 for the Per Protocol (PP) population]. The 90% CI was [−1.252 to 1.100] being within the pre-defined equivalence margin of ±3.0 g/d. Similar findings were observed in the Full Analysis Set (FAS) and the sensitivity analysis. Regarding the secondary outcomes, no differences over the intervention period were shown between the two groups in both the PP and FAS analysis sets. Average *Z*-scores were in the normal range based on World Health Organization (WHO) growth standards for both groups at all time points in both analysis sets. Stool consistency, amount, and colour were different in the two groups. No differences were observed in gut comfort, stool frequency, and formula intake, between the two groups. In total 14 AEs and 22 serious adverse events (SAEs) were reported of which 15 (12%) and 1 (5%) were considered as (possibly) related to the study product, respectively.

**Conclusions:**

The study demonstrates that the consumption of pHF results in adequate infant growth, equivalent to that of infants consuming IPF. Furthermore, the overall gut comfort was comparable between the two groups. Therefore, it can be concluded that the pHF is safe for and well tolerated by healthy infants.

**Clinical Trial Registration:**

https://clinicaltrials.gov/study/NCT05757323?id=NCT05757323&rank=1, identifier (NCT05757323).

## 1 Introduction

The World Health Organization (WHO) and the United Nations Children's Fund (UNICEF) recommend that infants are exclusively breastfed for the first six months of life to provide optimal nutrition in this critical period of life (1). For non-breastfed infants, infant formula is the most appropriate option to nourish the developing infant and according to recent reviews partially hydrolysed formulas are safe, well-tolerated, and lead to appropriate infant growth (2, 3). Still, the European Food Safety Authority (EFSA) emphasizes, at their most recent guideline, that the safety and suitability of each specific formula containing protein hydrolysates must be established by at least one adequately powered clinical study that evaluates measures of growth, in comparison to accepted growth standards and a control formula (4).

Protein hydrolysate-based formulas are mainly used to prevent or manage cow's milk protein allergy (CMPA) in non-exclusively breastfed infants. CMPA is the most common food allergy in children under five years of age (5), caused by an abnormal immune reaction to cow's milk protein (4). About 2%–5% of all newborns suffer from CMPA within the first year of life (6) while 5%–15% of infants show symptoms suggestive of CMPA (7). Several studies have demonstrated that the use of extremely hydrolysed protein fractions is effective in the management of CMPA in formula fed infants (8, 9). For less extensively hydrolysed protein fractions the risk reducing effect is not always clear and this should be established per product by scientific data. Potential additional benefits of partially hydrolysate-based products are suggested to be better taste, texture, and overall palatability in comparison to extremely hydrolysed formulas (10, 11).

Gastro-esophageal reflux, constipation, and colic are among the most common functional gastrointestinal disorders in infancy and early childhood (12). Most of these disorders are related to an underdeveloped gastrointestinal system. It is suggested that (partially) hydrolysed protein improves the gastrointestinal comfort in healthy term infants, especially in the early postnatal period (13).

The aim of the current study was to evaluate the weight gain of healthy infants consuming a partially hydrolysed whey protein-based infant formula (pHF) compared to a standard intact protein-based formula (IPF), over a period of at least three months (up until the age of 17 weeks). Secondary and tertiary objectives included evaluation of additional anthropometrics at specific timepoints over the period of three months, as well as assessment of gut comfort and stool characteristics between the two groups.

## 2 Material and methods

### 2.1 Study design and population

This study was a randomized, controlled, double-blind equivalence trial including two study arms; the pHF group and the IPF group. The study was conducted in healthy, full-term, exclusively formula-fed infants that were randomly allocated to receive one of the two formulas. The total duration of the intervention for each participant was at least three months, where a month was defined as 30 days (4). Specifically, three follow-up visits were performed in total, at the following time-points: 8 weeks of age (Follow-up 1), 13 weeks of age (Follow-up 2), and 17 weeks of age (Follow-up 3) with an allowed deviation of ±1 day. Recruitment was conducted between August 2021 and July 2022 at three sites in Greece, being the regional units of Attica, Thessaly, and Thessaloniki. The measurements were obtained during home visits by well-trained research associates: five medical doctors (MDs) in Attica, one MD and two nurses in Thessaly, and one midwife in Thessaloniki.

Infants were recruited until the 28th day of age by paediatricians in private practices. The paediatricians screened for interest and made the first contact with the parents/legal guardians at any moment from birth until 27 days of age. Paediatricians only approached parents/legal guardians of exclusively formula-fed infants and only the ones that showed interest were provided with a parent information leaflet to read at their convenience. After providing any additional clarifications and feedback to parents/legal guardians, those still interested to participate in the study were asked to sign the informed consent form. Only after signing, infants were screened on whether they fulfilled the inclusion criteria, and if the infant was confirmed to be eligible for participation in the study, the baseline measurements were performed during a home visit. In all cases parents/legal guardians were free to withdraw their infants from the study at any time without any consequences.

Inclusion criteria included: (i) full-term infants, (ii) healthy birthweight (between 2,500 and 4,200 g) according to WHO Child Growth Standards (14), (iii) boys and girls, (iv) healthy at birth and screening, (v) Weight-for-age *Z*-score (WAZ), weight-for-length *Z*-score (WHZ), and length-for-age *Z*-score (LAZ) at screening within the normal range according to WHO Child Growth Standards (i.e., between −2 and 2), (vi) age at enrolment ≤28 days of age, (vii) exclusively formula fed for at least 5 days prior to inclusion, (viii) exclusively formula fed during the entire intervention period, (ix) parents agreeing to initiate complementary feeding after finalization of the study, (x) being available for follow up until the age of 17 weeks, and (xi) written informed consent from parent(s) and/or legal guardian(s) aged ≥18 years. On the other hand, exclusion criteria included: (i) gestational age <37 weeks, (ii) birth weight <2,500 g or >4,200 g, (iii) age at enrolment >28 days, (iv) severe acquired or congenital diseases, mental or physical disorders, including CMPA, lactose intolerance and diagnosed medical conditions that are known to affect growth, (v) illness at screening/inclusion, (vi) incapability of parents to comply with the study protocol, (vii) illiterate parents, (viii) participation in another clinical trial, (ix) unwillingness to accept the formula supplied by the study as the only formula for their child during study participation, and (x) infants fed a special diet other than standard, non-hydrolysed, cow's or goat's milk based infant formula.

The study protocol, the parental information leaflet, and the informed consent form were reviewed and approved by Harokopio University Ethics Committee (approval code G2197/14-04-2021). The study was performed in accordance with the declaration of Helsinki (64th WMA General Assembly, Fortaleza, Brazil, October 2013). The trial was conducted in agreement with the International Conference on Harmonisation (ICH) guidelines on Good Clinical Practice (GCP), it was registered at the Netherlands Trial Register (identifier NL9536) and was published on clinicaltrials.gov as study ID NCT05757323.

### 2.2 Study procedures and formulas

Upon inclusion in the study, participants were randomized to one of the four coded products representing the two formulas. Randomization was performed centrally, at Harokopio University, based on schedules produced by means of SAS® Studio Version 9.4 M5, by using the procedure PROC PLAN. A block size of 8 was used, and stratification based on gender was implemented.

The entire study team, including the Principal Investigator (PI), co-Principal Investigators, the paediatricians, the clinical research associates and the Sponsor's Project Manager and Principal were blinded to the study formulae. An independent paediatrician was assigned to monitor adverse and serious adverse events (AEs and SAEs, respectively) during the trial and to evaluate whether de-blinding would be necessary.

Formulas were provided for free to the participating families during the scheduled home visits at every time point. Formula consumption was *ad libitum*, but a feeding table instruction was provided with the parental information leaflet to guide and encourage the appropriate for age consumption of the supplied products.

The nutritional composition of the two study products (produced by FrieslandCampina) is presented in Table 1 below. The macronutrient composition of the IPF (Frisolac 1) was similar to the composition of the pHF (Frisolac HA) apart from the protein fraction. The pHF contained 100% partially hydrolysed whey proteins. Both formulas complied with the compositional requirements laid down in Delegated Regulation 2016/127 (15). All formulas were provided in similar blank tins of 400 g each that carried the description “not for commercial use”. Four different codes were printed on the bottom of the tins to ensure blindness of the study. The entire study team as well as the participants were blinded to the study formulas. The treatment codes were revealed only after the trial was completed and the database had been locked.

**Table 1:** Composition of the study formulas (per 100 ml).

	pHF	IPF
Energy (kcal)	66	66
Protein (g)	1.6	1.4
Intact protein (g)	–	1.4
Protein hydrolysate (g)	1.6	–
Fat (g)ik	3.4	3.4
DHA (mg)	17	17
AA (mg)	6.9	6.9
Carbohydrates	7.1	7.2
GOS (g)	0.17	0.27

pHF, partially hydrolyzed whey protein infant formula; IPF, standard intact protein formula; DHA, docosahexaenoic acid; AA, arachidonic acid; GOS, Galacto-oligosaccharides.

During the baseline visit, a brief self-administered questionnaire was filled in by parents/legal guardians to obtain information on some socio-demographic indices and infant's and family members' medical history, alongside the baseline anthropometric measurements that included body weight, length, and head circumference. During each scheduled home visit, anthropometric measurements were performed in duplicate by the research associates, and the mean of two, or median of three measurements (when difference between the two measurements in body weight, length and head circumference was more than 20 g, 0.7 cm or 0.5 cm respectively) were reported. Furthermore, the Infant Gastrointestinal Symptoms Questionnaire (IGSQ) (16), which assesses overall gut comfort and incidence of minor digestive issues (e.g., vomits/regurgitation, colic, constipation, diarrhoea and crying episodes), was filled by the research associates who interviewed the parents/legal guardians. Lastly, the Amsterdam Infant Stool Scale (AISS) (17), a crying diary, and a formula intake diary, were filled by parents/legal guardians before each scheduled home visit and were handed to research associates upon the visits.

All (serious) adverse events [(S)AE] had to be reported by parents/legal guardians to study personnel including the paediatricians. The paediatrician in charge of the specific infant evaluated whether these events were AE or SAE and whether the event was (possibly) related or not to the study product. Furthermore, an independent paediatrician (medical advisor) was assigned to monitor SAEs during the trial, and to evaluate whether de-blinding would be necessary.

### 2.3 Primary, secondary and tertiary outcome variables measured

The primary outcome was weight gain (g/day) from baseline until 17 weeks of age, calculated as the difference in infant weight between the baseline visit and at 17 weeks of age, divided by the exact number of days between these two visits. Secondary outcomes included weight (kg), length (cm), head circumference (cm), and body mass index (BMI; kg/m^2^) at baseline and at each follow-up visit, alongside their respective *Z*-scores, calculated using the WHO Anthro Survey Analyser (14). Tertiary outcomes included gastrointestinal comfort parameters (assessed via the IGSQ and a crying diary), stool characteristics (assessed via the AISS), formula intake, and safety parameters*.* More details on the primary, secondary, and tertiary outcome measures can be found in Supplementary Table S1.

### 2.4 Sample size

The sample size calculation was based on the t-test for equivalence testing of two independent groups. For the margin of equivalence, a weight gain of ±3.0 g/day was used, which is considered to be nutritionally relevant (18). A standard deviation (SD) of 6.0 g/day was used based on the Scientific Committee on Food report from 2003 (5), which recommends that infant growth studies should have the power to detect a difference in weight gain equal to 0.5 SD. The randomization ratio between the pHF and IPF groups was 1:1.

The sample size required for establishing equivalence was calculated with SAS Studio (version 9.4 M5), with the procedure PROC POWER, assuming a mean difference in weight gain between the pHF and IPF groups of 0.9 g/day, a SD of 6.0 g/day, a significance level of 5%, and a power of 80%. This resulted in 103 infants per treatment group. Assuming 25% of randomized infants would be excluded from the per protocol (PP) analysis set, a minimum of 138 enrolled infants per treatment group was required (total of *n* = 276).

A pre-planned interim statistical analysis was performed by an independent statistician, to check the assumptions for the sample size calculation, including the data of (first enrolled) 121 participants. Based on the outcome of the interim analysis, it was advised to the study team to increase the sample size to a total of 345 participants.

### 2.5 Statistical analysis

All statistical analyses were performed by independent biostatisticians (OCS Life Sciences), using the SAS® software version 9.4 M5 or higher (SAS® Institute, Cary, North Carolina). For all analyses (unless otherwise stated), a two-sided statistical significance level of *α* = 0.05 was used. No correction for multiplicity was done because there was only one primary comparison of interest. Drop-outs of the study were not replaced, and missing data were not imputed. Additional sensitivity analyses were performed excluding any outlying observations in order to confirm the robustness of the statistical analyses.

The primary outcome, weight gain per day (g/day) between baseline and week 17, was analysed using a parametric growth curve (PGC) mixed effect model with an Huynh-Feldt variance-covariance matrix. Study formula, time (age in days) and time*time were added as fixed effects. Study formula and gender were included as fixed interaction terms with the time effect (formula*time, formula* time*time, gender*time, and gender*time*time). Gender, weight at birth and maternal gestational diabetes were included as covariates. The primary outcome was analysed in the FAS and PP analysis sets, with the PP analysis considered primary.

Length gain (cm/day) and weight gain (g/day) at weeks 8, 13 and 17 were analysed using the PGC mixed effect model including study formula, time (age in days), and time*time as fixed effects, and formula*time, formula*time*time, gender*time, and gender*time*time as fixed interaction terms, adjusting for the covariates gender and weight at birth (and maternal gestational diabetes when applicable), with a heterogeneous Toeplitz variance-covariance matrix or a Huyn-Feldt variance-covariance matrix. Head circumference gain (cm/day) at weeks 8, 13 and 17 (relative to baseline) was analysed with an Arbitrary Means Model (AMM) including study formula and time (categorical visit) as fixed factors, and formula*time and gender*time as fixed interaction terms, adjusting for covariates gender and weight at birth, with an unstructured variance-covariance matrix.

Length (cm), weight (g), head circumference (cm), and BMI (kg/m^2^), were analysed using a Mixed Model Repeated Measurements (MMRM) analysis with study formula and visit as fixed factors and gender, and birth measurements as covariates. The impact of factors that may influence the intervention effect (such as site and maternal gestational diabetes), were evaluated and added as covariates when applicable.

## 3 Results

### 3.1 Study population

**Figure 1:**
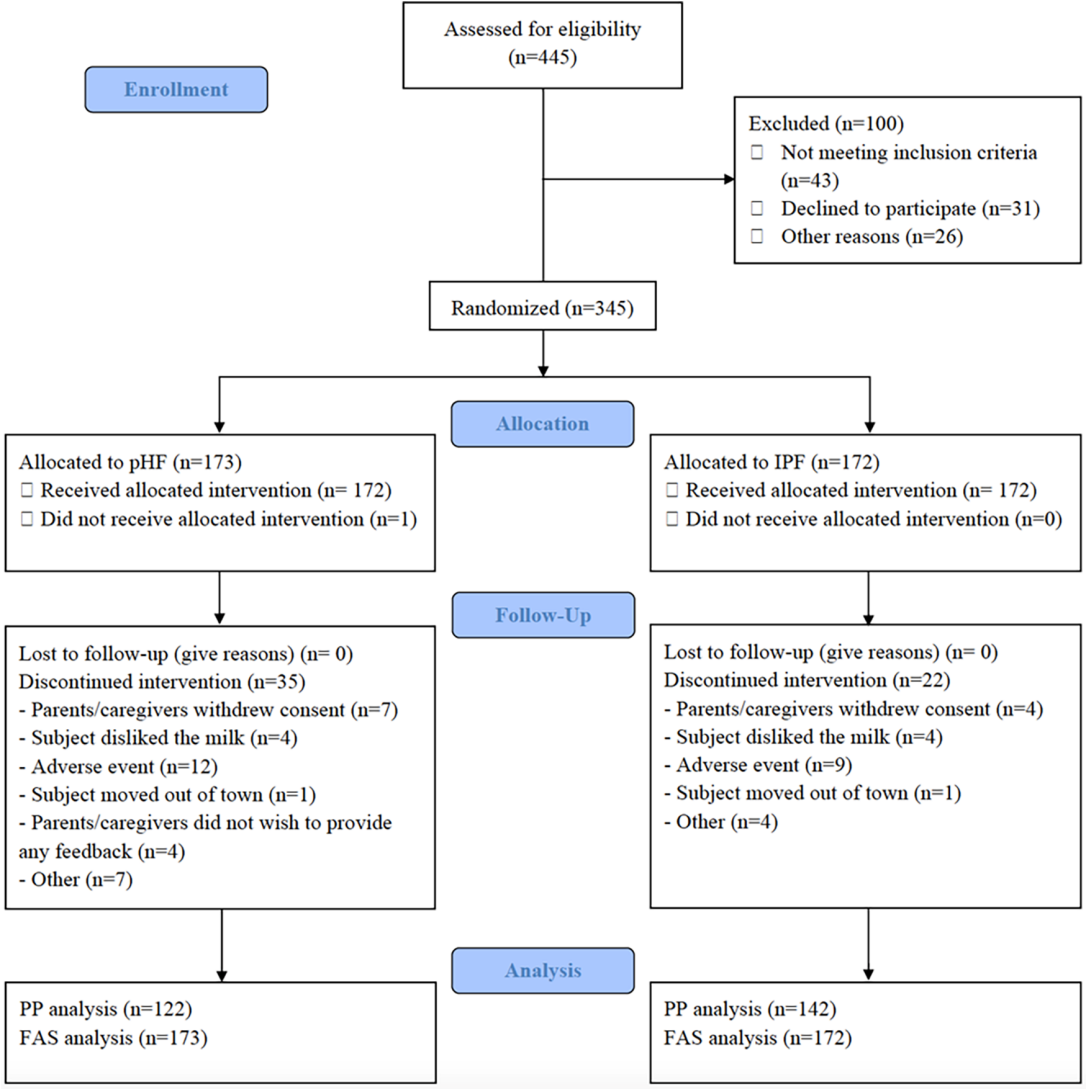
Study flowchart and participants’ disposition. PHF, partially hydrolysed protein-based infant formula; IPF, standard intact protein-based formula; PP, per protocol; FAS, Full Analysis Set.

A total of 345 infants were enrolled in the trial and randomized to a treatment arm; 172 were fed with the pHF, 173 with the IPF. Of the 345 infants recruited, 288 infants completed the study, and had the last follow-up visit at 17 weeks of age (138 pHF, 150 IPF) while 57 infants (35 pHF, 22 IPF) discontinued the study prematurely. The reasons for discontinuation for each study group is shown in Figure 1.

The demographic, perinatal and birth characteristics of study participants for the PP analysis set are presented in Table 2. From the total 264 participants, 158 were male and 106 were female, all born in Greece. Most demographic, perinatal and birth characteristics were well balanced between the study groups. Prevalence of maternal gestational diabetes was found to be somewhat higher in the pHF group compared to the IPF group (18.0% vs. 12.6%) in the full analysis set (FAS) (data not shown), but not for the PP set.

**Table 2:** Demographic, perinatal and birth characteristics of study participants and their parents.

			PP analysis set	
			pHF (*N* = 122)	IPF (*N* = 142)
Infant characteristics				
Gestational age (weeks)		Mean (SD)	38.3 (1.1)	38.3 (0.9)
Birth weight (g)		Mean (SD)	3,159.4 (382.4)	3,208.0 (366.3)
Birth length (cm)		Mean (SD)	50.1 (1.7)	50.1 (1.8)
Birth head circumference (cm)		Mean (SD)	34.2 (1.2)	34.3 (1.3)
Gender, male		*n* (%)	74 (60.7)	84 (59.2)
Number of live born infants from the pregnancy	1	*n* (%)	118 (96.7)	137 (96.5)
	2	*n* (%)	4 (3.3)	5 (3.5)
Caesarean section	Yes	*n* (%)	89 (73.0)	90 (63.4)
Study site	Attica	*n* (%)	84 (68.9)	91 (64.1)
	Thessaly	*n* (%)	26 (21.3)	34 (23.9)
	Thessaloniki	*n* (%)	12 (9.8)	17 (12.0)
Maternal characteristics				
Maternal age (years)		Mean (SD)	33.3 (6.0)	31.8 (5.7)
Maternal BMI at screening (kg/m^2^)		Mean (SD)	27.2 (5.6)	26.9 (5.1)
Maternal gestational diabetes	Yes	*n* (%)	22 (18.0)	15 (10.6)
	No	*n* (%)	100 (82.0)	125 (88.0)
	Unknown	*n* (%)	0 (0.0)	2 (1.4)
Maternal education level	<6 years	*n* (%)	4 (3.3)	7 (4.9)
	7–9 years	*n* (%)	6 (4.9)	6 (4.2)
	10–12 years	*n* (%)	27 (22.1)	32 (22.5)
	13–14 years	*n* (%)	32 (26.2)	39 (27.5)
	15–16 years	*n* (%)	32 (26.2)	29 (20.4)
	>16 years	*n* (%)	21 (17.2)	29 (20.4)
Maternal smoking during pregnancy		*n* (%)	28 (23.0)	26 (18.3)
Paternal characteristics				
Paternal age (years)		Mean (SD)	36.2 (6.5)	35.2 (6.7)
Paternal BMI (kg/m^2^)		Mean (SD)	28.5 (5.6)	27.5 (4.1x)
Paternal education level	<6 years	*n* (%)	5 (4.1)	10 (7.0)
	7–9 years	*n* (%)	11 (9.0)	11 (7.7)
	10–12 years	*n* (%)	46 (37.7)	58 (40.8)
	13–14 years	*n* (%)	25 (20.5)	24 (16.9)
	15–16 years	*n* (%)	19 (15.6)	17 (12.0)
	>16 years	*n* (%)	15 (12.3)	20 (14.1)
Paternal smoking during pregnancy		*n* (%)	18 (14.8)	19 (13.4)

Anthropometric baseline characteristics between the IPF and the pHF group in the PP and FAS sets were similar, except for head circumference-for-age and BMI-for-age *Z*-score, which were lower in the pHF group compared to the IPF group in the PP analysis set.

### 3.2 Weight gain and growth

The daily weight gain (g/d) from baseline to 17 weeks of age of the pHF and IPF group was found to be equivalent for the PP population (mean of 30.9 g/d [95% CI: 29.8, 31.9] vs. 30.9 [95% CI: 30.0, 31.9] as calculated with the PGC model), with a difference in estimated means of −0.076 [90% CI of (−1.252, 1.100)]. Similar results were found for the FAS analysis set (data not shown) where the mean difference between the study groups was −0.718 [90% CI of (−1.803, 0.368)]. The sensitivity analyses for both PP and FAS sets also showed equivalence in weight gain from baseline to 17 weeks of age of the two groups (mean difference was −0.116 with a 90% CI of [−1.391, 1.158] and 0.095 with a 90% CI [−1.102, 1,292]; data not shown). No significant differences were found for gains in length (cm/day) and head circumference (cm/day) at any follow up visit (week 8, week 13, and week 17) (*p* > 0.05) for both study groups in the PP and FAS populations (data not shown).

**Figure 2:**
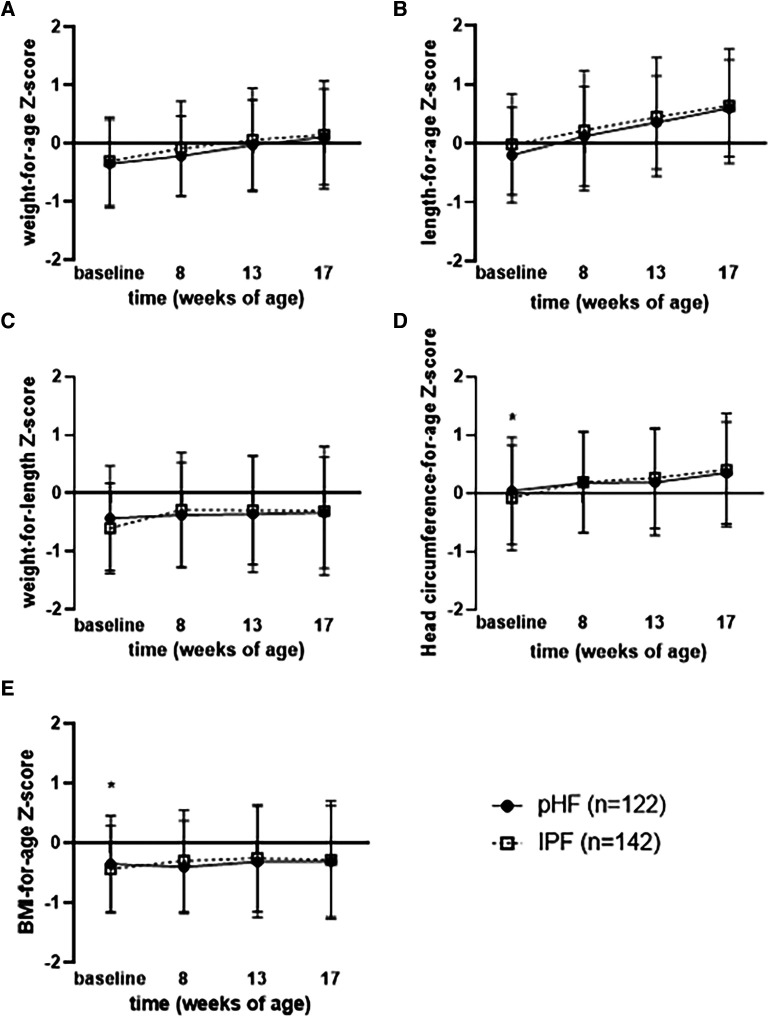
Mean (±90% CI) (**A**) weight for age, (**B**) length for age, (**C**) weight for length, (**D**) head circumference for age, and (**E**) BMI for age wHO growth standard *z*-scores per visit for pHF and IPF groups.

Regarding the secondary outcomes, no significant differences between the two groups were found in the PP analysis set for weight (g), length (cm), head circumference (cm), BMI (kg/m^2^), as presented in Table 3. In line with that, no differences were found for weight-for-age, length-for-age and weight-for-length *Z*-scores at any time point (presented in Figures 2A–C) or over the entire intervention (no overall effect over time (*p* > 0.05, Supplementary Table S1) between the study groups. Although BMI-for-age and head circumference-for-age *Z*-scores at baseline were different between groups [difference of means pHF vs. IPF (90% CI): 0.155 (0.032, 0.277) and 0.202 (0.045, 0.358) respectively], no difference was observed at any later time point (presented in Figures 2D–E). Furthermore, there was no average effect over the entire intervention period for both BMI-for-age (*p* = 0.607) and head circumference-for-age *Z*-scores (*p* = 0.377). In the FAS analysis set (Supplementary Table 2), no significant differences were observed between the two groups for any growth parameter at any time point (week 8, week 13, week 17), as well as no overall effect over the entire intervention period (*p* > 0.05).

**Table 3:** Anthropometric measurements of study participants of the PP population (mean ± SD), analysed with a mixed model repeated measurements model.

Outcome parameter	Visit (at age of)	pHF	IPF	*p*-value
Weight (g)^a^	Baseline	3,875.5 ± 483.4	3,900.1 ± 452.1	0.784
	Week 8	5,126.3 ± 521.5	5,206.4 ± 547.2	0.580
	Week 13	6,164.7 ± 649.0	6,226.9 ± 673.1	0.799
	Week 17	6,842.9 ± 734.4	6,874.9 ± 756.1	0.878
Length (cm)^b^	Baseline	52.9 ± 1.8	53.3 ± 1.9	0.106
	Week 8	57.6 ± 1.9	57.8 ± 2.0	0.503
	Week 13	61.5 ± 1.8	61.7 ± 2.1	0.512
	Week 17	64.3 ± 1.9	64.3 ± 2.1	0.877
Head circumference (cm)^c^	Baseline	36.4 ± 1.2	36.3 ± 1.2	0.145
	Week 8	38.8 ± 1.1	38.8 ± 1.1	0.780
	Week 13	40.4 ± 1.2	40.4 ± 1.1	0.848
	Week 17	41.6 ± 1.2	41.6 ± 1.3	0.821
BMI (kg/m^2^)^d^	Baseline	13.8 ± 1.1	13.7 ± 1.0	0.284
	Week 8	15.4 ± 1.1	15.6 ± 1.2	0.538
	Week 13	16.3 ± 1.4	16.4 ± 1.3	1.000
	Week 17	16.6 ± 1.4	16.7 ± 1.4	0.967

^a^ The mixed model repeated measurements model included study formulae and visit as fixed factors, gender, weight at birth, study site as covariates with a variance components variance-covariance matrix.

^b^ The mixed model repeated measurements model included study formulae and visit as fixed factors, gender, length at birth, study site as covariates with an autoregressive variance-covariance matrix.

^c^ The mixed model repeated measurements model included study formulae and visit as fixed factors, gender, head circumference at birth, maternal gestational diabetes, and study id as covariates with an autoregressive variance-covariance matrix.

^d^ The mixed model repeated measurements model included study formulae and visit as fixed factors, gender, BMI at birth, study site as covariates with a variance components variance-covariance matrix.

### 3.3 Formula intake

No difference was observed in average daily formula intake between the pHF and IPF groups at any time point of the study period (*p* > 0.05), as is presented in Table 4.

**Table 4:** Average daily formula intake (ml/day) at each follow-up visit by study group [mean (SD)].

Visit	pHF (*n* = 172)	IPF (*n* = 173)	*p*-value*
Week 8	783.5 (167.2)	807.7 (174.3)	0.130
Week 13	848.8 (195.6)	853.6 (173.1)	0.486
Week 17	900.8 (179.1)	905.9 (180.0)	0.863

* For continuous variables, *p*-values were calculated using the individual sample *t*-test for normally distributed data. For not-normally distributed data, *p*-values were calculated using a Wilcoxon-Mann-Whitney test.

### 3.4 Gut comfort and stool characteristics

**Figure 3:**
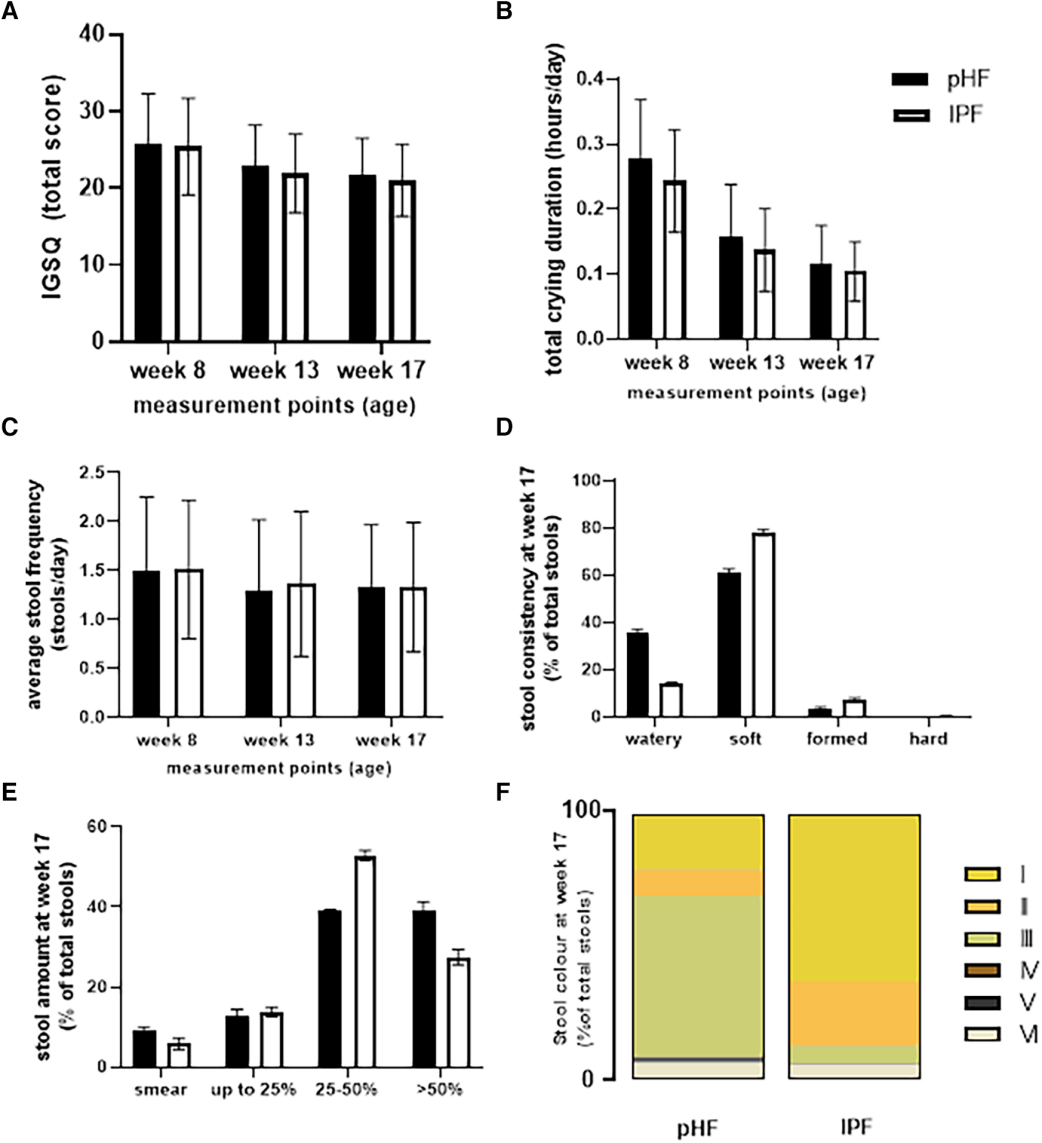
Average (**A**) IGSQ, (**B**) daily crying duration, and (**C**) stool frequency, (**D**) consistency, (**E**) volume, and (**F**) colour assessed by the AISS in the pHF and IPF groups.

The average IGSQ score, total daily crying duration, and daily stool frequency and characteristics as assessed by the AISS for the two groups are presented in Figure 3. No differences were found between the two groups in the total IGSQ scores (Figure 3A). Similarly, no differences were observed in average daily crying duration between the two groups, which decreased over time during the study period (Figure 3B). Regarding stool frequency and stool characteristics, no differences were found between the study groups on the daily frequency of stools (Figures 3C–F). As presented in Figure 3D, mean stool consistency scores at week 17 of age were significantly different between the two groups. This was consistent throughout the intervention period (*p* < 0.001, data not shown). For the pHF group, watery stools were reported more frequently, while for the IPF group, soft and formed stools were reported more frequently. Almost no hard stools were reported during the study for either group. Stool amount was not different at week 17 of age (Figure 3E), but was found to be significantly different between the two groups at weeks 8 and 13 (*p* < 0.001, data not shown). For the pHF group score 4: >50% of the diaper was reported more frequently compared to the IPF group. Stool colour was significantly different throughout the intervention between the two groups. For the pHF group, mainly the green colour was reported, followed by yellow, while for the IPF group most stools were reported as yellow or orange, as is shown in Figure 3F for the week 17 visit.

### 3.5 Safety parameters

Overall, 124 AEs occurred in the study population, 54 of them occurred in the pHF group and 70 occurred in the IPF group (data not shown). The majority of the AEs (88%) were judged by the paediatricians as unrelated to the intervention and 15 (11 in the pHF group and 4 in the IPF group) were judged as (possibly) related (AEs concerned: posseting (*n* = 4), vomiting (*n* = 3), other gastrointestinal symptoms (*n* = 5), strong odor (*n* = 1), crying (*n* = 1) and decreased appetite (*n* = 1). In total, 22 infants (13 in the pHF group and 9 in the IPF group) discontinued the study due to an AE and 4 infants (3 in the pHF group and 1 in the IPF group) due to a SAE (data not shown). One SAE was judged as possibly related to the pHF; in this case the subject was admitted at the hospital by the parents due to a colic event, and based on the hospital's policy, an overnight stay was applied.

## 4 Discussion

The findings of the present multicenter, randomized clinical trial confirmed equivalence in daily weight gain of healthy non-breastfed infants, fed with either a partially hydrolysed whey protein-based infant formula (pHF) compared to a standard intact protein-based formula (IPF) until the age of 17 weeks, over an intervention period of at least three months. Furthermore, all other growth parameters (weight, length, head circumference and BMI) assessed during the study period were similar between the two groups, while the respective *Z*-scores were in the normal range according to the WHO growth standards for both groups (14). Gut comfort assessment showed no differences in total IGSQ scores and daily crying duration between the pHF and IPF groups, while both outcomes decreased over the intervention period from baseline for both groups. Stool frequency was similar between the pHF and IPF groups; however, stool characteristics differed significantly, with the pHF group reporting more watery stools compared to soft or firmed stools which were reported more frequently in the IPF group.

The current results for growth outcomes, including the primary outcome of daily weight gain, are in agreement with findings from previous studies. First and foremost, similar results were found in the study by Karaglani et al., which assessed the effects on growth parameters of a pHF compared to an IPF in 163 healthy formula-fed infants over a period of three months (19). Similarly, other previous studies investigating the effects of different pHFs on growth indices of infants as compared to intact protein-based formulas or breast milk have found no differences among the study groups (20–23). Moreover, during the 10-year follow-up of the German Infant Nutritional Intervention Study (GINI) no differences were observed in weight, length and BMI gains of infants with atopic heredity fed with either a partially hydrolysed formula, an extensively hydrolysed formula, a cow's-milk based formula or breast milk (24, 25). All these findings collectively suggest that infant formulas containing partially hydrolysed cow's milk proteins are safe and support normal growth of infants from birth. However, as infant formula manufacturing processes and protein hydrolysates used may vary, each specific product still needs to be evaluated by clinical data. Also, each specific infant formula's nutritional composition is different and may contain various supplemental ingredients which may play a role in infant growth.

Regarding gut comfort, both groups in the current study showed good overall gastrointestinal outcomes with similar trends of improvement (decrease in total IGSQ scores and daily crying duration) over the intervention period. Comparable results were found in the study by Wu and colleagues that showed no significant gastrointestinal tolerance difference among healthy infants who consumed either a formula based on hydrolysed whey protein combined with intact casein protein—or intact protein-based formula or were breastfed (21). Vivatvakin et al. who studied 256 healthy infants also found no difference in the mean scores of IGSQ between infants fed with a pHF or an IPF, while lower mean total scores were observed with increasing age of the infants (26). As the control groups of the above-mentioned studies showed similar findings as the test products, this might indicate that the improvement shown over time is not related to the formulas, but that other factors played a role. An explanation could be the development of the gastrointestinal tract of the infants that matures with age. For example, peaks in crying are around 6 weeks after birth and strongly decreases from the age of 12 weeks onwards (27).

Although no difference was observed in the stool frequency of the two groups in the current study, there were some differences in stool characteristics. For the IPF group most stools were reported to be soft, which might be attributed to the addition of the prebiotic galacto-oligosaccharides (GOS; 2.7 g/L) (28). For the pHF group also most stools were reported to be soft, however higher number of stools with a more watery consistency were observed compared to the IPF, despite the lower levels of added GOS in the pHF (1.7 g/L). For comparison, watery stool consistency has been reported for breastfed than formula fed infants during the first three months of life (29). While the stool colour in the IPF group was mostly yellow or orange, the stools of the pHF group were mostly green and some were yellow. Similarly, to the current findings, Picaud et al. also found no differences in stool frequency of infants in the pHF group compared to the IPF group during an intervention period until the age of 17 weeks. In addition, similar stool consistency was found between the two groups except for a slightly higher percentage of watery stools observed in the pHF group at 17 weeks of age (23). Kuehn et al. observed softer mean stool consistency in the infants fed with whey-casein pHF compared to the infants fed with a commercial non-hydrolysed whey-casein formula (20). So, several clinical studies have now indicated that consumption of partially hydrolysed proteins by healthy infants may affect stools, and result in a lower stool consistency score compared to a formula containing intact protein only. Furthermore, the protein source seems to affect the stool colour, of which the clinical relevance needs further investigation.

To our knowledge, this study is one of a few well-designed, double-blinded, randomized clinical trials in healthy term infants that explore the effects of an infant formula containing partially hydrolysed whey protein on growth, gut comfort and stool characteristics compared to a standard intact cow's milk protein-based formula. Among the strengths of the current study is that everyone involved in it, from parents/legal guardians to researchers and statisticians, were blinded to the formula products. Furthermore, the pre-planned and accordingly performed interim analysis to check sample size assumptions and thus sample size adequacy was of great importance. The interim analysis outcome advised to increase sample size (from total *n* = 276 to *n* = 345) for sufficient study power for comparative analysis. Another major strength is the fact that the nine research associates who collected the data at the three study sites were well-trained, used the same standardized equipment, and followed the same standardized procedures. Lastly, having different study sites within the country where the study was conducted contributes to the representativeness of the study results. Although in this study there were no indications for it, one possible limitation is the collection of parent/legal guardian-reported data on formula intake, stool characteristics and crying duration, that are subjective and may lead to under- or over- reporting and require a high degree of cooperation; albeit such data are difficult to be obtained in a more objective way for such a long intervention period. The lack of a breastfeeding group as a reference in this study could be considered as another potential limitation, although the aim of the study was to investigate the growth of formula-fed infants.

## 5 Conclusions

In summary, the results of the current study demonstrated equivalent growth outcomes between the pHF and the standard IPF in healthy infants until 17 weeks of age, supporting adequate growth in accordance with the WHO growth standards. Moreover, gut comfort parameters were comparable between the two groups. Some differences were observed in stool consistency, colour and volume. In conclusion, the pHF examined is safe and suitable to support adequate growth in, and is well-tolerated by, healthy term infants.

## Data Availability

The datasets presented in this article are not readily available because the datasets contain personal data including data from medical records. Data sharing without participants’/guardians’ consent specifically about sharing such data beyond specific research purposes and the research team could breach participants’ confidentiality. The generated datasets may be available upon request to the corresponding author depending on the purpose of the request.
