# Supplementary material for: Growth and gut comfort of healthy term infants exclusively fed with a partially hydrolysed protein-based infant formula: a randomized controlled double-blind trial
Source: Front Pediatr. 2024 May 17;12:1328709. doi: 10.3389/fped.2024.1328709 (PMC11141394; doi:10.3389/fped.2024.1328709)
Supplement: Supplementary file 1 [file Datasheet1.pdf]

Supplementary Material

1 Supplementary Data

The primary outcome of the study was weight gain per day between baseline and at 17 weeks of age, calculated as the difference in infant weight between the baseline visit and at 17 weeks of age, divided by the exact number of days between these two visits. Secondary outcomes included weight (kg), length (cm), head circumference (cm), and BMI (kg/m^2) at baseline and at each follow-up visit, alongside weight-for-length, weight-for-age, length-for-age, BMI-for-age and head circumference-forage Z-scores, measured and calculated at each follow-up visit according to the guidelines of World Health Organization (WHO) Anthro. Tertiary outcomes included gut comfort (Infant Gastrointestinal Symptom Questionnaire (IGSQ), crying diary, Amsterdam Infant Stool Scale (AISS~~)~~), formula intake, and safety parameters.

Body weight, length and head circumference were measured two times by the research associates at every scheduled visit, according to the methods described in a Standard Operating Procedure (SOP). All measurements for one subject were performed and recorded by the same research associate who recorded the measurements in the relevant form. If the difference between the two measurements in body weight, length and head circumference was more than 20 g, 0.7 cm, or 0.5 cm, respectively, an additional measurement was performed in the respective indices. If the second measure exceeded the difference limit, then a third measurement was taken. The final value was based on the mean of two, or median of three measurements.

Body weight was reported in grams, using a digital scale appropriate for infants with a precision of ±20 g for weights below 20 kg. Care was taken to place the scale on a flat, stable surface and every effort will be made by the research associate to ensure that restless infants are kept calm during the weighing procedure. All children were weighed undressed, with a clean and dry diaper, without jewellery or other ornaments and preferably before feeding. Furthermore, the research assistants calibrated the digital scales monthly, as specifically detailed in the relevant SOP.

Recumbent length was reported in centimetres and measured to the nearest 0.1 cm using an infantometer, with a stationary head piece making sure to align infant's head in the vertical Frankfurt plane, a sliding vertical foot piece and a horizontal back piece with a measure tape mounted on it. All children were measured undressed, with a clean and dry diaper and without ornaments. Care was taken to place the infantometer on a flat and stable surface and every effort was made by the research associates to ensure that restless infants are kept calm during the measurements. BMI was calculated by dividing weight (in kg) with length squared (in m^2). WHO Anthro for personal computers, version 3.2.2, 2011: Software for assessing growth and development of the world's children, was used to generate Z-score values for weight-for-age, length-forage, weight-for-height and BMI-for-age.

Head Circumference (HC) was reported in centimeters and measured to the nearest 0.1 cm with the use of a non-elastic tape and with the infant at a sitting position. More specifically, HC was measured to the nearest 0.1 cm and passing the measure tape around the head, just above the eyebrows, above the ears on each side and over the occipital prominence at the back of the head to its maximal

circumference. WHO Anthro for personal computers, version 3.2.2, 2011: Software for assessing growth and development of the world's children was used to generate Z-score values for head circumference-for-age.

The Infant Gastrointestinal Symptoms Questionnaire (IGSQ) was used to assess infant's overall gut comfort and minor digestive issues (i.e., vomits/regurgitation, colic, constipation, diarrhoea and crying episodes). The questionnaire was filled in by the research associates during an interview with the parents at each scheduled home visit. To get more insight in overall comfort, crying was determined. A crying diary was used to assess hours of crying per day. Parents were asked to fill in the crying diary at home during the three days before the scheduled follow-up visit by the research associates, recording the sleeping, awake and crying periods (of more than 5 minutes each time) during the entire day (a day = 24 hrs; i.e., for 72 hrs in total). In addition, the AISS was used to assess the stool consistency (four categories: watery, soft, formed and hard), amount/volume (smear to more than 50% of the nappy's surface) and colour (six categories) of stools, as described elsewhere. The AISS was filled in by parents at home whenever their infant defecated in the three days before the scheduled visit by the research associate.

The timing and frequency of feeding occasions and the exact amount/volume (in mL) of formula consumed by infants (i.e., volume of formula prepared – volume of left-over formula) at each feeding occasion was recorded in diaries over a period of seven consecutive days before the follow up visits. Explicitly during the first month of study participation, parents also completed the formula intake diary at day 3 up until 7, and at day 10 up until 14 after baseline. Any occasion of vomiting or regurgitation was recorded as well. The research associates contacted the parents by phone to retrieve the formula intake data from the first two weeks of the intervention. The diaries were collected by the research associates during their follow-up home visits. Parents also had to hand-in the emptied tins as a measure of compliance. When non-compliance with the protocol was observed (i.e., any type of mixed feeding either by combining breastmilk or other type(s) of formula or food with the test products), these infants were excluded from the PP analysis set.

An Adverse Event (AE) was defined as any untoward medical occurrence in a subject (infant) which did not necessarily have to have a causal relationship with the study formula or study procedures. An AE could therefore be any unfavorable and unintended sign (including an abnormal laboratory finding, for example), symptom, or disease temporally associated with the use of the study products, whether or not considered related to the products used. A Serious Adverse Event (SAE) was defined as any AE, adverse reaction, or unexpected adverse reaction, respectively, that i) resulted in death, ii) was lifethreatening*, iii) required hospitalization or prolongation of existing hospitalization, iv) resulted in persistent or significant disability or incapacity, v) consisted of a congenital anomaly or birth defect.

AEs and SAEs that parents reported to the paediatricians were recorded by the paediatrician using the relevant (S)AE form. SAEs had to be reported to the Principal Investigator within 24 hours from the moment the paediatrician was informed on the SAE. Regarding SAEs, the Principal Investigator had to notify the Sponsor and the independent paediatrician (responsible for independently monitoring SAEs) within another 24 hours from the moment he was informed on the SAE. SAEs were coded by ICD/MedDra classification.

** The term life- threatening in the definition of a SAE refers to an event in which the participant has a risk of death at the time of the event. It does not refer to an event which, hypothetically, might have caused death if more severe.*

1.1 Supplementary Tables

Table 1: Anthropometric measurements of study participants of the PP population (mean±SD).

Outcome parameter	Formula Difference pHF vs. IPF<br>Estimate (90% CI)	p-value
Weight (g)[1]	-5.884 (-92.542, 80.774)	0.911
Length (cm)[2]	-0.158 (-0.436, 0.119)	0.348
Head circumference (cm)[3]	0.039 (-0.131, 0.210)	0.703
BMI (kg/m^2)[4]	0.018 (-0.178, 0.214)	0.882
Weight for age z-score[5]	0.023 (-0.100, 0.145)	0.761
Length for age z-score[6]	-0.016 (-0.094, 0.062)	0.739
Weight for length z-score[7]	0.023 (-0.130, 0.176)	0.805
Head circumference for age z-score[8]	0.080 (-0.069, 0.228)	0.377
BMI for age z-score[9]	0.044 (-0.097, 0.185)	0.607

**pHF = protein-based infant formula; IPF = intact protein-based infant formula; BMI = body mass index.*

[1]The mixed model repeated measurements model included study formula and visit as fixed factors, gender, weight at birth, study site as covariates with a variance components variance-covariance matrix.

[2]The mixed model repeated measurements model included study formulae and visit as fixed factors, gender, length at birth, study site as covariates with an autoregressive variance-covariance matrix.

[3]The mixed model repeated measurements model included study formulae and visit as fixed factors, gender, head circumference at birth, maternal gestational diabetes, and study id as covariates with an autoregressive variance-covariance matrix.

[4]The mixed model repeated measurements model included study formulae and visit as fixed factors, gender, BMI at birth, study site as covariates with a variance components variance-covariance matrix.

*[5]The arbitrary means model included study formulae and categorical time as fixed factors, formulae*time and sex*time as fixed interaction terms, adjusting for gender, weight at birth, maternal gestational diabetes, and study site as covariates with an unstructured variance-covariance matrix.*

*[6]The arbitrary means model included study formulae and categorical time as fixed factors, formulae*time and sex*time as fixed interaction terms, adjusting for gender, weight at birth, maternal gestational diabetes, and study id as covariates with a variance components variance-covariance matrix.*

*[7]The arbitrary means model included study formulae and categorical time as fixed factors, formulae*time and sex*time as fixed interaction terms, adjusting for gender, weight at birth, and study site as covariates with a unstructured variance-covariance matrix.*

*[8]The arbitrary means model included study formulae and categorical time as fixed factors, formulae*time and sex*time as fixed interaction terms, adjusting for gender, weight at birth, maternal gestational diabetes, and site id as covariates with a Huynh-Feldt variance-covariance matrix.*

*[9]The arbitrary means model included study formulae and categorical time as fixed factors, formulae*time and sex*time as fixed interaction terms, adjusting for gender, weight at birth, maternal gestational diabetes, and study site as covariates with an antedependence variance-covariance matrix.*

Table 2: Anthropometric measurements of study participants of the FAS population (mean±SD).

Outcome parameter	Visit (at age of)	pHF	IPF	p-value
Weight (g)[1]	Baseline	3828.2 (470.5)	3857.2 (466.8)	0.894
	Week 8	5118.8 (518.3)	5183.4 (541.7)	0.554
	Week 13	6148.2 (645.2)	6196.6 (680.6)	0.694
	Week 17	6849.5 (722.8)	6861.3 (753.5)	0.756
Length (cm)[2]	Baseline	52.98 (2.00)	53.15 (1.98)	0.282
	Week 8	57.79 (2.02)	57.73 (2.11)	0.878
	Week 13	61.63 (2.03)	61.64 (2.19)	0.843
	Week 17	64.38 (2.03)	64.35 (2.18)	0.918
Head circumference (cm)[3]	Baseline	36.32 (1.19)	36.26 (1.29)	0.220
	Week 8	38.75 (1.10)	38.72 (1.12)	0.775
	Week 13	40.36 (1.22)	40.37 (1.18)	0.920
	Week 17	41.58 (1.21)	41.61 (1.25)	0.873
BMI (kg/m^2)[4]	Baseline	13.61 (1.14)	13.61 (1.05)	0.606
	Week 8	15.31 (1.14)	15.54 (1.20)	0.224

	Week 13	16.18 (1.37)	16.29 (1.31)	0.617
	Week 17	16.51 (1.36)	16.55 (1.44)	0.942

**pHF = protein-based infant formula; IPF = intact protein-based infant formula; BMI = body mass index; FAS: full analysis set.*

[1]*The mixed model repeated measurements model included study formulae and visit as fixed factors, gender, weight at birth, maternal gestational diabetes as covariates with a variance components variance-covariance matrix.*

[2]*The mixed model repeated measurements model included study formulae and visit as fixed factors, gender, length at birth, maternal gestational diabetes as covariates with an autoregressive variance-covariance matrix.*

[3]*The mixed model repeated measurements model included study formulae and visit as fixed factors, gender, head circumference at birth, maternal gestational diabetes as covariates with an autoregressive variance-covariance matrix.*

[4]*The mixed model repeated measurements model included study formulae and visit as fixed factors, gender, BMI at birth, study site as covariates with a variance components variance-covariance matrix.*
